# Integrating mixed reality preparation into acute coronary syndrome simulation for nursing students: a single-group pretest-posttest study

**DOI:** 10.1186/s12912-024-02110-9

**Published:** 2024-07-09

**Authors:** Sun-Hee Moon, Hyeonjin Jeong, Mi Jin Choi

**Affiliations:** 1 College of Nursing, Chonnam National University, Gwangju, South Korea; 2 Clinical Research Center, Chonnam National University Hwasun Hospital, Hwasun, South Korea; 3 College of Nursing, Gyeongsang National University, Jinju, South Korea

**Keywords:** Mixed reality, Immersive simulation, Technology, Acute coronary syndrome, Nursing education, Assessment, Clinical competence

## Abstract

**Background:**

Timely and effective intervention within the ‘golden hour’—the critical first 90 min after the symptom onset—is crucial for initiating life-saving treatment and reducing mortality in acute coronary syndrome (ACS). This highlights the need for nursing students to be proficient in ACS care, emphasizing the importance of preparatory training. This study enhanced traditional simulation methods by integrating a mixed reality (MR) preparation step, offering a more immersive learning experience. We aimed to evaluate the effectiveness of integrating MR preparation into ACS simulation education, focusing on enhancements in knowledge, self-confidence in learning, and self-efficacy in learning. Additionally, we examined performance, practice immersion, and satisfaction to comprehensively evaluate the MR application.

**Methods:**

One-group pretest-posttest design was implemented in a convenience sample of thirty-nine senior nursing students from a university in South Korea in August 2022. We developed a simulation program integrating MR preparation into ACS simulation (IMRP-ACSS), which was validated through expert review for content validity. The students participated in the simulation program over six hours across two days, including a 40-minute individual session of MR-based simulation preparation using head-mounted displays (the HoloLens 2). Individual changes in knowledge, self-confidence in learning, and self-efficacy in learning evaluated by the survey were analyzed using paired t-tests. Additionally, group performance assessed using the checklist was analyzed. Immersion and satisfaction were measured with a tool and a 10-point Likert scale, respectively.

**Results:**

Individually, participants demonstrated significantly increased knowledge (*t* = 11.87, *p* < .001), self-confidence in learning (*t* = 7.17, *p* < .001), and self-efficacy in learning (*t* = 4.70, *p* < .001) post-education. Group performance yielded a mean score of 56.43/70 ± 7.45. Groups scored higher in electrocardiogram interpretation, patient safety, and heparin administration. Participants reported a practice immersion level of 37.82/50 ± 9.13 and expressed satisfaction with the program, achieving an average score of 8.85/10 ± 1.35.

**Conclusion:**

Integrating MR preparation into ACS simulation enhanced nursing students’ knowledge, self-confidence in learning, and self-efficacy in ACS care, providing a replicable and immersive learning experience. This method is an effective addition to nursing education, preparing students through comprehensive, technology-enhanced training.

**Supplementary Information:**

The online version contains supplementary material available at 10.1186/s12912-024-02110-9.

## Background

Globally, cardiovascular diseases are a leading cause of mortality, with half of cases attributed to ischemic heart disease [1]. Acute coronary syndrome (ACS), a significant cardiovascular disease, can have varying health outcomes and prognosis depending on the management quality [2]. Timely and effective intervention within the ‘golden hour’— the critical first 90 min between a hospital visit and coronary artery ballooning intervention—is crucial for initiating life-saving treatment and reducing mortality in acute coronary syndrome (ACS) [3, 4], highlighting the importance of prompt and effective intervention [5–7]. It is essential to avoid any delays in the diagnostic process, which includes differential diagnosis with other causes such as psychogenic chest pain, 12-lead electrocardiogram (ECG) with interpretation, and serum troponin measurement, to ensure prompt treatment within the golden hour [5]. Additionally, healthcare providers should be familiar with the characteristics of ACS patients and the necessary treatment to be able to begin immediate management [5, 8].

Nurses play a key role in assessing patients’ condition at the bedside, providing them with the opportunity to promptly detect any critical changes. The care for ACS patients requires focused assessment and interventions guided by clinical judgment and decision-making. Clinical judgment is required for performing specific tests, such as ECG, and interpreting these results to recognize a cardiac arrest, leading to a delay in management and a diminished capacity to prevent patient deterioration [9-, 10]. Therefore, preparing nurses with the necessary knowledge and skills to perform accurate assessment, exercise clinical judgment, make appropriate decisions, and implement essential interventions in ACS patient care is vital in nursing education.

Nursing education through direct nursing experience in high-risk situations, such as ACS, is limited [9]. This can prevent nurses from fully developing the practical skills and judgment required in actual clinical settings [10]. Therefore, practical education that can enhance nursing capabilities in a safe learning environment to prevent failure to rescue before being involved in direct patient care is essential [11]. Education through experience in providing nursing care in diverse situations is imperative, surpassing mere acquisition of knowledge and skills. Hence, simulation can be a suitable nursing education method. In the current ACS nursing education, the simulation has shown positive results in knowledge, skills, and self-efficacy [12, 13]. In ACS nursing education, various studies underscored the efficacy of simulation-based training across multiple dependent variables. High-fidelity simulations are notably effective in enhancing the knowledge and practical skills of nursing students [12]. Furthermore, simulated patient methods significantly improve nurse self-efficacy compared to traditional lecture-based approaches [13]. Additionally, simulations incorporating high- and low-fidelity mannequins, as well as professional actors, positively impact knowledge acquisition and confidence of healthcare emergency professionals, including nurses [14]. Given the diverse levels of experience and knowledge among different target groups, the application and effectiveness of simulation-based education for ACS may vary. This variability must be carefully considered in educational design and implementation. Including a dedicated preparation phase in simulation training is crucial, particularly for nursing students, who often have limited practical experience with ACS patients. This preparatory phase is essential to bridge knowledge gaps and ensure that all students are adequately equipped to effectively manage such high-stakes clinical scenarios. By integrating mixed reality (MR) as a preparatory tool, we aimed to bridge the knowledge and skill gaps before students engage in high-stakes, high-fidelity simulations. This approach not only reinforces knowledge and practical skills but also boosts self-efficacy, culminating in a more effective simulation learning experience. This methodology aligns with the INACSL Healthcare Simulation Standards of Best Practice™, particularly the 2021 Prebriefing standards, which emphasize the critical role of thorough simulation preparation in achieving successful learning outcomes [15]. However, the traditional simulation methods utilizing human patient simulators (HPSs) and standardized patients (SPs) require a substantial allocation of educational resources, including instructors, incurring significant costs [16]. Thus, implementing repetitive education is challenging, introducing the possibility of varying educational quality depending on instructors and their availability. Education using technologies such as virtual reality (VR) and MR have recently been introduced into nursing simulation to improve the above-mentioned educational limitations. Simulations using VR and MR might be expensive during initial development but are advantageous due to repetitive and step-by-step training, as well as increased immersion [17]. Therefore, it is necessary to develop an appropriate method by considering the pros and cons of nursing education simulation.

MR is defined as the merging of the real and virtual worlds, primarily considered a technology that transparently overlays the virtual environment onto the real world using a head-mounted display (HMD) [18, 19]. MR can be suitable for healthcare education in various contexts as it provides situational and authentic experiences connected to the real environment and enhances the interaction between physical and virtual elements, preserving a sense of presence [20]. In healthcare education, MR has been applied to knowledge acquisition in anatomy and skill training for surgeries or complex procedures, outperforming traditional learning approaches [17, 21, 22]. Simulation using MR is a new educational tool that combines the advantages of VR and augmented reality (AR) [23]. MR simulation can offer an immersive experience of actively participating in complex clinical settings, serving as an alternative to the traditional clinical practice environment [16, 23]. MR can provide feedback on skills and knowledge, stimulate the learning process, and improve learners’ competency through immersive scenarios [17]. In nursing education, MR has been applied to nursing simulations for critical situations and skill acquisition, such as CPR, pain assessment for myocardial infarction, patient monitoring in intensive care setting, nasogastric tube care, and coping with fire situations [24–28]. MR has great potential to enhance nursing simulation education, particularly in high-risk, low-frequency scenarios, such as ACS patient care. This area urgently needs innovative approaches, such as MR simulation preparation interventions, to provide nursing students with the essential competencies required for effective management. Moreover, there is a growing demand for the development and evaluation of tailored MR integrating simulation in nursing education [17, 23, 29]. MR integrating simulation can serve as an effective and efficient educational approach for ACS care, enhancing nurses’ performance by integrating clinical judgment and skill acquisition through immersive experience and real-time interactions.

According to the Layered Learning Theory presented by Bauman [30, 31], the effectiveness of education can be maximized when traditional didactic education, digital-based simulation experience, and mannequin-based simulation experience are connected to real-world experience. In this context, MR-based simulation corresponds to the second stage of this theory, the digital-based simulation. Most nursing simulations, including existing ACS simulations, primarily focused on utilizing and verifying the effectiveness of single-modality simulations, traditionally using HPS or SP and, more recently, incorporating VR or MR. Therefore, we require a stepwise approach for nursing simulation education while incorporating digital simulations such as MR to enhance the effectiveness of simulation education and increase efficiency through repeated education. Consequently, we developed an ACS nursing simulation program comprising a series of educational stages, including theoretical education using video, simulation utilizing MR, and mannequin simulation. The primary objective of this study was to evaluate the effectiveness of integrating MR preparation into ACS simulation (IMRP-ACSS) education, focusing on enhancements in knowledge, self-confidence in learning, and self-efficacy in learning. Additionally, we aimed to assess the usability of MR as a preparatory tool in simulation training, specifically examining formative evaluations of performance, practice immersion, and satisfaction.

## Methods

### Design and sample size

This study utilized a single-group pretest-posttest design to examine the efficacy of integrating the IMRP-ACSS for nursing students [32]. The sample size calculation, performed with G*Power 3.1.9.7 software (Heinrich Heine University Düsseldorf, Düsseldorf, Germany), was predicated on a two-tailed paired t-test with a 5% significance level and 80% power, incorporating an anticipated effect size of 0.47. This effect size was adopted from a meta-analysis evaluating the knowledge outcomes of VR training in nursing education as we inferred that the immersive nature of MR, similar to VR, would produce a comparable effect in knowledge acquisition among nursing students [33]. The calculation yielded a minimum required sample size of 38 participants.

### Setting and participants

This study was conducted at a university, which provides undergraduate and graduate nursing education courses in a city, South Korea. One class of 89 senior nursing students was asked to participate during August 2022. Senior students at this university completed learning on assessment and intervention for ACS patients under the subjects of health assessment in the second year and medical-surgical nursing in the third year. However, these students did not participate in ACS-specific simulation scenarios before this study. Regarding prior simulation experiences, these students engaged in basic procedural simulations using simulators, such as intravenous fluid infusion and basic life support. Additionally, some students had experience with VR, standardized patients, and online simulations through extracurricular programs. None of the students previously engaged in MR simulations before participating in this study.

The intervention and data collection were implemented in August 2022. An educational notice was uploaded to an online chat including senior nursing students. Interested students were asked to complete a pre-survey developed by the faculty, using a Google Form. This pre-survey assessed their knowledge, self-confidence in learning, and self-efficacy in learning. Following their participation in the two-day IMRP-ACSS, the students completed a post-test survey. This survey repeated the initial items and included additional questions on education satisfaction and practice immersion.

Out of 49 participants who completed this pre-survey, 10 were excluded from the final analysis. Five were excluded due to inability to attend the intervention sessions because of COVID-19 infections or job interviews. The other 5 participants completed the intervention but were excluded due to no response to the post-survey despite repeated contact by the investigators. Therefore, data from the remaining 39 participants were analyzed.

### Development of an educational program

**Program development**. The program content requirements were established through a comprehensive review of literature and interviews conducted by our research team with students, professors, and clinical nurses, focusing on ACS-related educational needs. Additionally, a literature review on MR integrating simulations was performed to integrate relevant contents and functional requirements into the program development. Educational stages were structured in alignment with the Layered Learning Theory [30, 31].

**Expert validation**. The developed simulation scenario and educational materials underwent a content validity check by a panel of experts comprising four nursing professors, each with > 7 years of clinical experience in intensive care and emergency settings and expertise in simulation education. The content validity index (CVI) for the educational materials and simulation scenario was 1.00 and 0.96, respectively. Following expert feedback, educational materials were refined to emphasize ACS characteristics. Furthermore, oxygen saturation levels were adjusted in the simulation scenarios.

**Mixed reality display in the simulation**. The finalized ACS simulation used MR technology. An artificial intelligence (AI) voice actor was used to insert lines into a video depicting a patient with chest pain symptoms [34]. Audio and video of the patient monitor were recorded to enhance fidelity. Step-by-step skill acquisition videos, including operational procedures of an infusion pump, were also filmed. These components were integrated using Microsoft Dynamic 365 software (solution version 900.1.0.1), designed for HoloLens 2. Using Dynamics 365 on HoloLens 2 allows to recognize the learner’s hand and eye movements, real space, and pop-up 3D objects, as well as implement algorithms. We applied the quiz algorithm for each step, utilized 3D models provided by the software (such as 3D lines and 3D arrows), and uploaded pre-made photos and videos to the software to be played.

#### Education stages

**Fig. 1:**
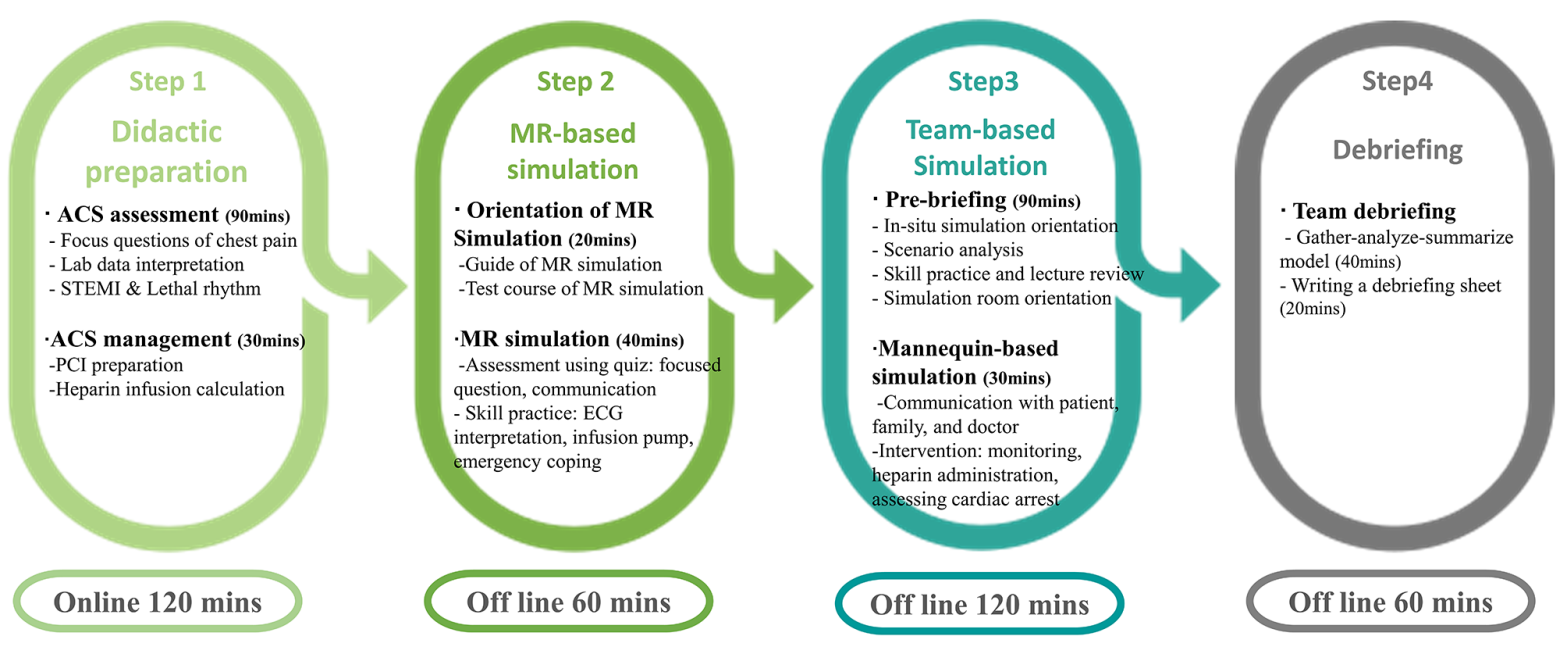
Four steps of integrating mixed-reality preparation into acute coronary syndrome simulation. Abbreviations: ACS, acute coronary syndrome; STEMI, ST elevation myocardial infarction; PCI, percutaneous coronary intervention; MR, mixed reality

We developed the IMRP-ACSS for nursing students that included four steps (Fig. 1). ***The first step included didactic lectures*** on ACS patient nursing. Participants studied seven online lectures loaded in the online learning system of the university for 120 min. The 90-minute lecture topics included focus questions regarding chest pain, interpretation of clinical laboratory data (troponin-I), 12-lead ECG analysis, and lethal rhythm recognition, while the 30-minute lecture topics included percutaneous coronary intervention (PCI) preparation, drug dose calculation, and heparin infusion method. Lectures were conducted by the first author, who has > 10 years of emergency department clinical experience and > 6 years of teaching experience.

***The second step comprised an individual MR-based simulation preparation***, utilizing the Microsoft HoloLens 2. This step aimed to evaluate participants’ ability to assess symptoms, interpret ECG, administer appropriate nursing care, and respond to emergent situations involving a virtual ACS patient depicted in the scenario (Supplementary Fig. 1). The ACS patient scenario was a patient described in the simulation book published by the first author [35]. The ACS patient in the scenario was a 72-year-old male who visited the emergency department by ambulance due to chest pain while walking in the park. ST elevation myocardial infarction (STEMI) was confirmed on a 12-lead ECG, and ventricular fibrillation occurred on the monitor while preparing for PCI. This scenario was integrated into the Microsoft HoloLens 2. Initially, participants undertook a 20-minute demonstration to familiarize themselves with the HoloLens 2. Subsequently, during a 40-minute individual session, they interacted with video simulations of patients experiencing chest pain, responded to 10 decision-making quizzes, and received immediate feedback on their answers. The participants practiced skills such as preparing medication and handling medical devices according to the virtual guide. The participants practiced coping with sudden chest pain, interpreting lethal rhythm, and decision-making for responding to cardiac arrest. No formal debriefing occurred at this stage, except for the feedback provided after the quiz. The researcher’s intervention was minimal, addressing only operational issues with the HoloLens 2 or answering students’ questions. There was no cybersickness among the participants.

***The third step involved a team mannequin-based simulation of ACS patient care***, continuing with the same patient scenario introduced in the previous MR simulation step. In contrast to the individual-focused MR simulation, this step emphasized collaboration and effective communication within the team and with physicians, patients, and caregivers to manage patients with ACS. Participants underwent a 90-minute pre-briefing session during which they analyzed the patient scenario, including medical history, vital signs, and initial orders, establishing team roles and familiarizing themselves with the clinical environment and equipment. The team mannequin-based simulation was conducted using Laerdal’s Resusci Anne®, a medium-fidelity simulator designed for practicing IV insertion and CPR, providing a realistic clinical environment enhancing the fidelity of the simulation. Teams, composed of 5–6 members, interacted with operators assuming the roles of patients, family members, and doctors. These scenarios necessitated the use of SBAR (situation, background, assessment, recommendation) communication techniques, which are critical for effective information exchange and clinical scenario management [36]. The first author, with four years of experience in conducting simulation classes, played various roles within the simulation to facilitate a dynamic and responsive learning environment. During the 30-minute team mannequin-based, participants were required to perform multiple tasks simultaneously, dividing roles among those who communicated directly with patients and their caregivers, those responsible for executing nursing interventions based on medical orders, and those tasked with documentation.

***The fourth step was the debriefing***, which was conducted in the simulation room immediately after the simulation. Over 40 min, the instructor asked questions to remind the participants of the situation according to the GAS (gather-analyze-summarize) debriefing model and encouraged participants to analyze the scenario and promote critical thinking and reflection [37]. This was followed by a 20-minute team debriefing where each group prepared a summary sheet, reviewed the simulation events, and formulated strategies for future clinical practice.

### Measures

#### Measures for individual outcomes

##### Knowledge

We developed a knowledge tool of 20 items in which participants could respond with true/false/unknown to statements related to knowledge on ECG and nursing interventions for ACS. The same panel of experts that assessed the content validity of the educational materials also verified the validity of this knowledge tool. The item-CVI values for the 20 items ranged from 0.50 to 1.00. According to Lynn [38], when 3–5 experts participate in the CVI, items with a CVI score < 1.0 are considered for deletion; however, items can be retained if appropriately modified based on experts’ feedback. Following this principle, we deleted two questions with an I-CVI value of 0.50 and modified the wording of two questions with an I-CVI value of 0.75 based on experts’ advice. The final knowledge tool consisted of 18 items, with a CVI of 0.93. The knowledge level was calculated by summing the number of correctly answered items out of 18. A high score reflects a deep understanding and comprehension of ACS nursing care.

##### Self-confidence in learning

The tool for measuring self-confidence was developed by the National League for Nursing and translated by Yoo [39, 40]. This tool comprises eight items rated on a 5-point Likert scale, where higher scores indicate higher learner confidence. The Cronbach’s alpha value of the original tool was 0.87, while the Cronbach’s alpha value of the translated tool was 0.72. In the current study, the Cronbach’s alpha value was 0.74. A high score denotes a strong confidence among participants in their ability to apply ACS nursing knowledge.

##### Self-efficacy in learning

We used a self-efficacy tool developed by Ayres and modified by Park and Kweon [41, 42]. This tool consists of 10 items rated on a 7-point Likert scale. The Cronbach’s alpha value of the original tool was 0.94, while the Cronbach’s alpha value of the modified tool was 0.95. In the current study, the Cronbach’s alpha value was 0.97. A high score indicates strong self-efficacy in learning and applying ACS nursing skills.

##### Satisfaction

Education satisfaction was assessed using a single item on a 10-point Likert scale developed by the faculty, where higher scores indicate higher levels of satisfaction. A high score suggests excellent satisfaction with the IMRP-ACSS.

##### Practice immersion

The flow short scale developed by Engeser and Rheinberg was simplified and translated by Yoo [42, 43]. It comprised ten items, with six items in the subdomain of performance proficiency and four items in the subdomain of performance immersion. A 5-point Likert scale was used, where a higher total score indicated a higher level of immersion. The translated scale demonstrated a Cronbach’s alpha of 0.84, while the Cronbach’s alpha in this study was 0.92. A high score indicates a profound level of engagement and involvement in the simulation experience.

#### Measures for group performance

***Group performance***: A checklist for performance evaluation for this ACS simulation was developed by modifying the checklists of the ‘heparin anticoagulation therapy scenario’ and the ‘nursing scenario for bradycardia/tachycardia treatment’ from the Nursing Department of Seoul National University Hospital [44]. In this study, the item CVI was 0.75–1.00, and the CVI for the entire tool was 0.90. According to Lynn [38], eight items in our study that initially registered an item-CVI of 0.75 were not removed. Instead, we revised their wording based on experts’ suggestions to enhance clarity and ensure relevance, thus maintaining their inclusion in the tool without compromising the validity of the instrument. The checklist comprises 20 items distributed across 7 subdomains (ECG interpretation, patient safety, heparin administration, infusion pump use, focused assessment, SBAR communication, and patient education), with each subdomain containing 2–3 items. Each item was scored on a 0/1/2 scale: 0 indicates that the task was not performed at all, 1 indicates partial performance, and 2 indicates complete performance. Scores within each subdomain were summed and normalized to a 10-point scale to facilitate a balanced comparison across the subdomains. This normalization was essential given the varying number of questions per subdomain. The total possible score for the checklist, representing the sum of the normalized scores from each subdomain, could range from 0 to 70 points. A high score indicates superior performance in ACS patient care within a team setting.

Inter-rater reliability was established through a structured training process for the evaluators involved in the study. Initially, the primary researcher and another trained evaluator independently scored two simulation videos using the performance checklist (weighted kappa = 0.67). They reviewed their scoring discrepancies, discussed each checklist item in detail, and reached a consensus to ensure uniformity in applying the evaluation criteria. In the group simulation, the evaluator assessed the team mannequin-based simulation performance during the third stage from a simulation control room equipped with a one-way mirror, utilizing the ACS nursing performance checklist.

### Statistical analysis

Participant characteristics are reported as means and standard deviations (SDs) for continuous variables and numbers and percentages (%) for categorical variables. The results of outcomes were reported as ranges, means, and SDs. A paired t-test was used to compare the levels of knowledge, self-confidence, self-efficacy, and satisfaction with education between pre- and post-education. SPSS statistics software version 27 (IBM Institute, NY, USA) was used to perform statistical analysis. Statistical significance was defined as a two-sided *P* value < 0.05.

## Results

The data of 39 participants were analyzed, excluding 10 who did not complete the program participation or post-education survey. Table 1 shows the general participants’ characteristics. All participants had previous simulation experiences.

**Table 1:** General characteristics of participants (*N* = 39)

Characteristics	Subcategories	*n*(%) or mean ± SD
Gender	Female	34(87.2)
Age (year)	-	23.00 ± 3.05
Previous simulation experience (multiple answer)	SP simulation	5(12.8)
	VR/AR simulation	18(46.2)
	Online simulation	9(23.1)
Interest in online games	Yes	10(25.6)
	Average game time (hour/week)	4.40 ± 3.91

Abbreviation: SD; standard deviation, SP; standardized patient, VR; virtual reality, AR; augmented reality

Table 2 summarizes the comparison of knowledge, self-confidence in learning, and self-efficacy in learning before and after program participation. After participation, knowledge (*t* = 11.87, *p* < .001), self-confidence in learning (*t* = 7.17, *p* < .001), and self-efficacy in learning (*t* = 4.70, *p* < .001) significantly increased.

**Table 2:** Comparison of knowledge, self-confidence in learning, and self-efficacy in learning (*N* = 39)

Variables	Range	Pre	Post	t	*p*
Knowledge	0–18	8.85 ± 3.62	14.77 ± 2.22	11.87	< 0.001
Self-confidence in learning	8–40	30.74 ± 4.05	35.79 ± 3.17	7.17	< 0.001
Self-efficacy in learning	10–70	56.82 ± 10.88	64.41 ± 6.75	4.70	< 0.001

Table 3 summarizes the evaluation of performance, practice immersion, and satisfaction with education. The overall mean performance score was 56.43 ± 7.45 out of 70. Specifically, higher performance scores were observed in the following order: ECG interpretation, patient safety, and heparin administration. The participants provided positive evaluations of the program related to satisfaction and immersion. Participants reported a practice immersion level of 37.82 ± 9.13 out of 50, and expressed satisfaction with the education, achieving an average score of 8.85 ± 1.35 out of 10.

**Table 3:** The level of performance, practice immersion, and satisfaction with education

Variables	Range	Min	Max	Mean ± SD
**Performance total (** *N* ** = 7)**	0–70	44.17	65.00	56.43 ± 7.45
ECG interpretation	0–10	6.67	10.00	9.05 ± 1.31
Patient safety	0–10	5.00	10.00	8.57 ± 1.97
Heparin administration	0–10	5.00	10.00	8.21 ± 1.83
Infusion pump use	0–10	5.00	10.00	7.86 ± 1.73
SBAR communication	0–10	5.00	10.00	7.86 ± 2.67
Focused assessment	0–10	5.00	10.00	7.50 ± 1.44
Patient education	0–10	3.33	10.00	7.38 ± 3.02
**Practice immersion total (** *N* ** = 39)**	10–50	11.00	50.00	37.82 ± 9.13
Proficiency in performance	6–30	7.00	30.00	23.62 ± 5.32
Immersion in performance	4–20	4.00	20.00	14.21 ± 4.26
**Satisfaction with education (** *N* ** = 39)**	1–10	5.00	10.00	8.85 ± 1.35

Abbreviations: SD, standard deviation; ECG, electrocardiogram; SBAR, situation-background-assessment-recommendation

## Discussion

While there is a demand for effective simulation methods to enhance ACS nursing, traditional simulations have limitations, such as resource-intensive requirements and challenges in reproducing qualified educational experience. Moreover, the effectiveness of the single use of virtual technology in implementing ACS nursing simulations is uncertain. In response, we developed a progressive education program including MR-based simulation preparation to enhance integrated nursing competencies for ACS. The MR-based simulation preparation that we incorporated facilitated interaction between the real world and the virtual scenario, enabling learners to experience patient assessment, medication administration, and procedures while practicing clinical judgment and skill acquisition in a realistic context. This educational program showed positive effects on knowledge, self-confidence in learning, self-efficacy in learning, and performance related to ACS nursing using one-group pretest-posttest study design.

Previous studies that incorporated MR into other nursing simulations, although not directly related to ACS nursing, reported positive effects on patient assessment, decision-making, skill training, and situational coping ability [24–28]. MR applications in nursing education are diverse. For example, a study demonstrated that telepresence via devices such as Google Glass enhances the efficiency and effectiveness of CPR training for nurses by improving completion times and success rates [24]. Another study showcased MR’s utility in remote patient monitoring, which enhanced patient safety and reduced medical errors by providing real-time clinical support [26]. However, such applications require the instructor’s remote presence, which may limit direct interaction. Contrasting these studies, our MR application allows learners to independently navigate through customized ACS care scenarios without immediate instructor intervention. This autonomy is crucial in fostering deeper learning and skill acquisition. Unlike the generic scenarios used in previous research [25], our program involved developing tailored scenarios for ACS nursing care integrated with advanced MR technology, such as HoloLens 2 and Dynamics 365. This approach not only included comprehensive scenario development but also incorporated voice contents, and interactive patient monitoring, enhancing the immersive learning experience. Other studies have applied MR to specific nursing tasks, such as nasogastric tube care [27], and managing emergency situations like operating room fires [28], demonstrating MR’s effectiveness in technical skill training and situational management skills. Our research adds to this body of evidence by offering a holistic simulation experience that encompasses the ACS patient care—from assessment to intervention—thereby confirming the potential of MR application to significantly enhance nursing education.

MR application in simulations, through its distinctive features, has advantages compared to other types of simulation modalities. Our educational program effectively utilized these features to achieve educational outcomes and benefits. First, this study simultaneously utilized virtual algorithms, immediate feedback functions, and real mannequins to enhance real-time interaction [45], allowing students to experience clinical judgment based on the interpretation of abnormal ECG and skill acquisition based on instructional procedures for tasks such as preparing and administering heparin. These enhancements align with findings from another study that employed MR in simulations, which demonstrated that an interactive self-training methodology with step-by-step operational instructions and a supportive teaching system not only heightened students’ interest in skill training but also improved learning outcomes compared to traditional teaching methods [27]. Second, students could experience scenario situations while maintaining a sense of presence in the real world by utilizing real images in the same space and audio to create an immersive virtual world overlaid with real environments when scenarios were implemented. In this study, the students’ immersion level was reported at 3.78 ± 0.91 out of 5, which was higher than the immersion level of 3.21 ± 0.50 among senior students observed in simulation utilizing other high-fidelity simulators [40]. This observation is consistent with findings from other studies employing MR in simulations, where the video integration created a more realistic environment than could be achieved with mannequins alone [46]. Finally, students could experience a qualified and standardized simulation preparation by programming and utilizing MR developed and validated by experts in advance. This overcomes the limitations of the traditional simulations conducted with instructors in real time, which can change by instructors and circumstances and require significant input of time and effort while also offering the advantage of increasing proficiency through repetitive practice [16, 27]. Additionally, it is possible to provide a qualified experience allowing for immersion and interaction by combining real-world and virtual technologies beyond the limitations of traditional simulations or single use of VR or AR [28, 29].

Participants in our educational program, which integrates MR preparation into ACS simulation, reported a significant increase in their self-confidence in learning and self-efficacy in learning. In comparison, another study indicated that simulation-based training using standardized patients enhanced self-confidence in ACS clinical nursing more effectively than traditional lectures [12]. However, there is a concern that lacking genuine self-confidence and self-efficacy might cause hesitation in executing immediate, necessary actions in emergencies, such as those requiring CPR, despite having adequate knowledge and skills [47, 48]. The immersive experience provided by the MR-based simulation preparation, followed by team manikin-based simulation, significantly enhanced the acquisition of self-confidence and self-efficacy. Nevertheless, these results must be interpreted with caution, acknowledging the potential discrepancies between perceived self-confidence or self-efficacy and actual performance capabilities [49].

Additionally, in our study, performance was assessed in groups of seven, while knowledge, self-confidence in learning, and self-efficacy in learning were evaluated on an individual basis. This group evaluation was not meant to serve as an individual result but as part of an intermediate, formative evaluation within the simulation process aimed at preparing participants for the final simulation assessment post-debriefing. This approach aligns with the established INACSL’s standards for simulation-based formative assessments, which seek to provide timely feedback and foster improvement throughout learning [50].

In the context of effectively incorporating MR into simulation education, there is a demand for the development of a tailored educational program that can achieve specific educational objectives. Additionally, there is an issue concerning the lack of theory or evidence-based design and valid evaluation when MR is applied in simulation education [17, 23, 29]. This study tried to design a tailored simulation program integrating MR preparation in ACS nursing, ensuring its validity through a systematic approach, and objectively evaluate outcomes related to specific educational objectives. Previous studies on education for ACS nursing and MR utilization in nursing education, were reviewed to reflect user needs and the content and functional requirements based on evidence [17, 23–29, 51–53]. Based on Bauman’s Layered Learning theory, this educational program was structured in stages, and the validity of the educational contents and evaluation tools was ensured through expert group review [30, 31]. As a result, this educational program might be a harmonious combination of MR technologies and theoretical evidence suitable for specific learning objectives of enhancing comprehensive ACS nursing competencies. Considering the benefits of preparedness for low-frequency but high-risk situations and the burden of inputting considerable time and effort into real-time traditional simulation methods associated with such situations, systematically developed educational programs incorporating MR can serve as a good alternative educational method [16]. In the future, expanding research regarding the development and evaluation of educational programs for educational demands in different situations might confirm the applicability of MR in nursing simulation. For instance, various educational settings, especially those with an educational need for preparation related to low-frequency but high-risk situations, might consider prioritizing the application of MR and developing more situation-specific educational programs. Additionally, this study did not include the final stage of the Layered Learning Theory, which is real-world experience in the educational program. Future research is recommended to expand the educational program to actual clinical settings and examine its short- and long-term effects. Furthermore, patient education, focused assessment, and SBAR communication areas showed scores of 7.38–7.86 in this study, indicating relatively weaker performance in communication-related tasks with patients or other healthcare professionals. This could be attributed to the limited opportunities for practicing various communication reactions within the MR-based simulation preparation. In the future, developing advanced educational programs utilizing technologies such as AI integration, which can enhance communication skills by incorporating various communication reactions, is suggested.

This study had certain limitations. First, the program was applied to a small group of students from a single university and evaluated the effect without a control group. The inherent constraints of this one-group pretest-post-test design limit our capacity to conclusively attribute the observed changes solely to the program [32]. External factors such as concurrent educational activities or testing effects could also contribute to the results. Moreover, the sequential arrangement of the individual MR-based simulation preparation followed by the team mannequin-based simulation might have facilitated recall rather than independent decision-making, potentially biasing the performance outcomes. Second, the instruments and methods used for evaluation present limitations. The tools employed to assess knowledge, performance, and satisfaction were developed or adapted by our research team. Despite a rigorous revision process based on expert feedback, the validity of these instruments, particularly the single-item satisfaction scale, may still be questioned. Furthermore, although the initial calibration between raters achieved a weighted kappa value of 0.67 in the performance evaluation, indicating moderate agreement, the subsequent reliance on a single evaluator for the final assessments might introduce subjectivity into performance scores. Third, a 10% non-response rate further complicates interpretation, as reasons for dropout such as time constraints or survey fatigue remain speculative. This non-response rate could potentially bias the study findings and require cautious interpretation of the data.

## Conclusion

The integrating mixed reality preparation into acute coronary syndrome simulation contributed to improving students’ ACS nursing competencies. The integration of MR into ACS simulation education significantly enhanced the nursing students’ knowledge, self-confidence, and self-efficacy in learning. These findings substantiate the utility of MR as a powerful tool in preparing nursing students for real-world clinical challenges by providing immersive, real-time interactions within both realistic and virtual scenarios. When considering the demand for high-quality education and resource-saving in nursing simulation settings and the needs of students for active learning and performance capabilities related to coping with situations, MR-integrated simulation education can be used as an educational tool that allows students to experience satisfying and immersive learning, enhance integrated nursing performance capabilities, and increase self-confidence and self-efficacy. Further research is needed to develop and verify the effects of MR-integrated simulation education, which requires the interest and capacity development of educators in MR utilization and investment in MR application by institutions.

### Electronic supplementary material

Below is the link to the electronic supplementary material.

Supplementary Material 1

## Data Availability

We have made our research materials freely available on “Protocol IO” for anyone to download. The materials can be accessed at the following URL: https://www.protocols.io/blind/07B08F64E20D11EE999C0A58A9FEAC02.

## Abbreviations

ACS: Acute Coronary Syndrome
AR: augmented reality
BLS: basic life support
CPR: cardiopulmonary resuscitation
CVI: content validity index
ECG: electrocardiogram
GAS: gather-analyze-summarize
HPS: human patient simulator
IMRP-ACSS: integrating mixed reality preparation into acute coronary syndrome simulation
MR: mixed reality
SD: standard deviation
SP: standardized patient
STEMI: ST elevation myocardial infarction
VR: virtual reality
