# Supplementary figures and images for: Integrating mixed reality preparation into acute coronary syndrome simulation for nursing students: a single-group pretest-posttest study
Source: BMC Nurs. 2024 Jul 9;23:468. doi: 10.1186/s12912-024-02110-9 (PMC11232335; doi:10.1186/s12912-024-02110-9)

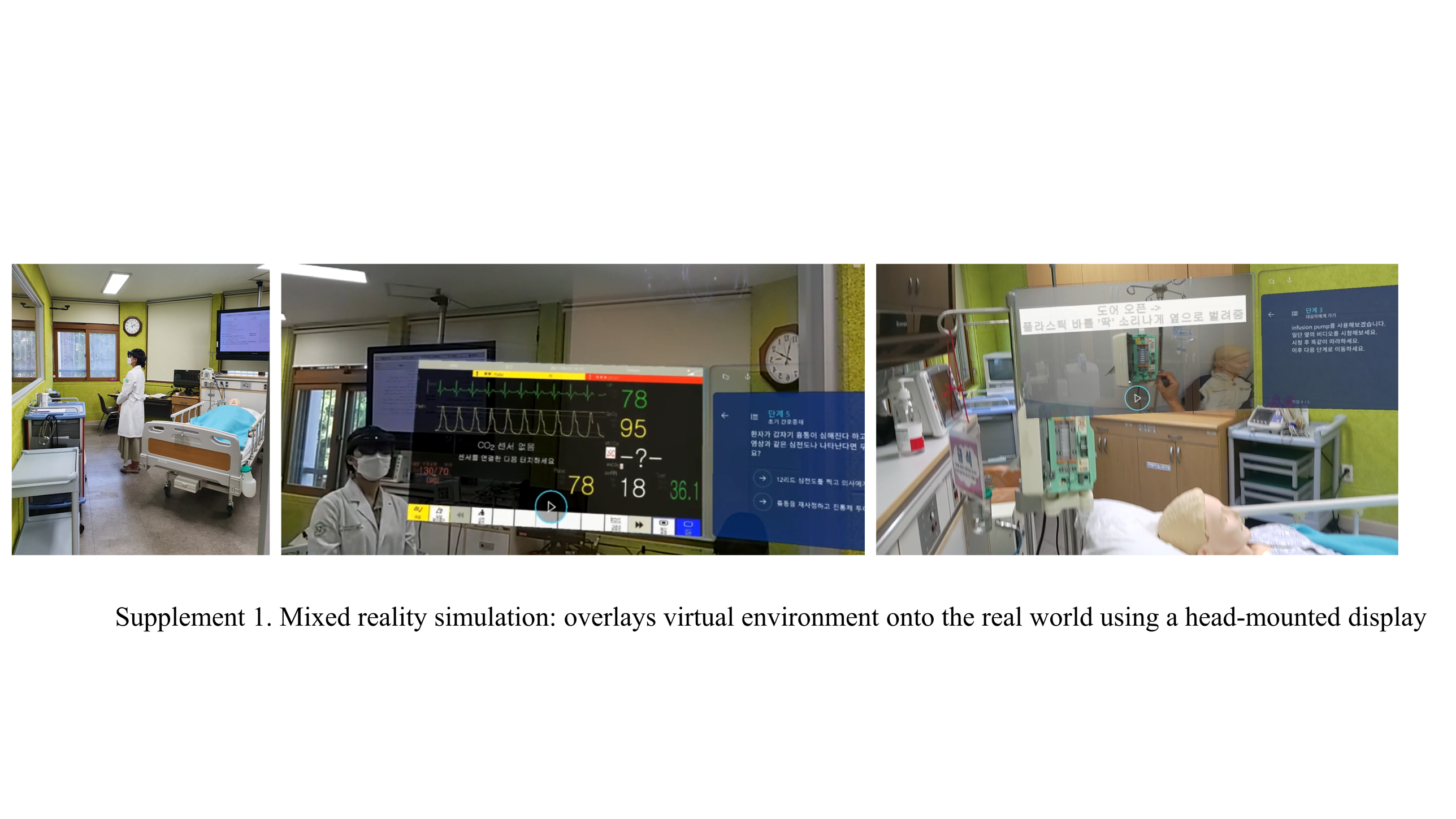

Supplement: Supplementary file 1 — Supplementary Material 1 [file 12912_2024_2110_MOESM1_ESM.tif]
